# 芯片液相色谱技术进展

**DOI:** 10.3724/SP.J.1123.2020.07031

**Published:** 2021-04-08

**Authors:** Hanrong WEN, Jue ZHU, Bo ZHANG

**Affiliations:** 厦门大学化学化工学院, 福建 厦门 361005; College of Chemistry & Chemical Engineering, Xiamen University, Xiamen 361005, China; 厦门大学化学化工学院, 福建 厦门 361005; College of Chemistry & Chemical Engineering, Xiamen University, Xiamen 361005, China; 厦门大学化学化工学院, 福建 厦门 361005; College of Chemistry & Chemical Engineering, Xiamen University, Xiamen 361005, China

**Keywords:** 液相色谱, 芯片色谱, 微流控芯片, 微型化, 综述, liquid chromatography (LC), microchip chromatography, microfluidics, miniaturization, review

## Abstract

微型化是现代分析仪器发展的重要趋势。微型化液相色谱仪器在提供与常规尺度液相色谱相同甚至更高分离效率的同时,可以有效减少溶剂和样品的消耗;在液相色谱-质谱联用中,低流速进样可以有效提高质谱离子源的离子化效率,提高质谱检测效率;对于极微量样品的分离,微型化的液相色谱可以有效减少样品稀释;液相色谱的微型化还有利于液相色谱仪器整体的模块化和集成化设计。芯片液相色谱是在微流控芯片上制备色谱柱并集成相应的流体控制系统和检测系统。芯片液相色谱是色谱仪器微型化的一种重要方式,受到学术界和产业界的普遍关注,但是这一方式也充满挑战。液相色谱微流控芯片需要在芯片基底材料、芯片色谱柱的结构设计、微流体控制技术、检测器技术等方面做出创新,使微流控芯片系统适配液相色谱分离技术的需要。目前芯片液相色谱领域面临的主要问题在于芯片基底材料的性质难以满足芯片液相色谱进一步微型化和集成化的需求;因此芯片液相色谱在未来的发展中需要着重关注新型微流控芯片基底材料的开发以及微流控芯片通道结构的统一设计。该文着重介绍了芯片液相色谱技术近年来的研究进展,并简要展示了商品化芯片色谱当前的发展情况。

## Abstract (en)

Miniaturization is an important trend in modern analytical instrument development, including miniaturized gas chromatography and liquid chromatography, as well as micro bore columns and capillary-to-microfluidics-based platforms. Apart from the miniaturization of the separation column, which is the core part of a chromatographic system, other parts of the system, including the sampler, pumping system, gradient generation, and detection systems, have been miniaturized. Miniaturized liquid chromatography significantly reduces solvent and sample consumption while providing comparable or even better separation efficiency. When liquid chromatography is coupled with mass spectroscopy, a low flow rate can increase the ionization efficiency, leading to enhanced sensitivity of the mass spectrometer. In contrast, normal-scale liquid chromatography suffers from its relatively high volumetric flow rate, which challenges the scanning frequency of the mass spectrometer. On the other hand because of the small sample size, other detection strategies such as spectrometric methods cannot provide sufficient sensitivity and limits of detection. In this sense, mass spectrometry has become the detection method of choice for micro-scale liquid-phase chromatography. Miniaturized liquid chromatography can diminish sample dilution efficiently when extremely small amounts of samples are used. The main driving force for this miniaturization trend, especially in liquid-phase separations, is the desperate need for microscale analyses of biological and clinical samples, given these samples are precious and the sample size is usually very small. At present, microscale liquid-phase chromatography is the only method of choice for such small, precious, and highly informative samples. The miniaturization of liquid chromatography systems, especially chromatographic columns, would be advantageous to the modularization and integration of liquid chromatography instrumental systems. Chip liquid chromatography is an integration of chromatography columns, liquid control systems, and detection methods on a single microfluidic chip. Chip liquid chromatography is an excellent format for the miniaturization of liquid chromatography systems, and it has already attracted significant attention from academia and industry. However, this attempt is challenging, and great effort is required on fundamental techniques, such as the substrate material of the microfluidic chip, structure of the micro-chromatography column, fluid control method, and detection methods, in order to make the chips suitable for liquid chromatography. Currently, the major problem in chip liquid chromatography is that the properties of the chip substrate materials cannot meet the requirements for further miniaturization and integration of chip liquid chromatography. The strength of the existing chip substrate materials is generally below 60 MPa, and the material properties limit further advances in the miniaturization and integration of chromatographic chips. Therefore, new chip substrate materials and the standard of chip channel design such as channel size and channel structure should be the key for further development of chip liquid chromatography. Mainstream instrumentation companies as well as new start-up innovation companies are now undertaking efforts toward the development of microchip liquid chromatographic products. Agilent, the first instrumentation company that introduced commercial microchip liquid chromatographic columns to the market, has led this field. Apart from microchip-based columns, Agilent introduced trap columns for different kinds of biological molecules as well as gradient generation systems for microchip-based liquid phase chromatography. Recently, another start-up company introduced microchip columns based on the *in situ* microfabrication of the column bed rather than packing the column with a particulate material. Such developments in microfabrication may further propel the advancement of micro-scale liquid-phase chromatography to an unprecedented level, which is beyond the conventional components and materials employed in normal-scale liquid chromatography. This review introduces the recent research progress in microchip liquid chromatography technologies, and briefly discusses the current state of commercialization of microchips for liquid chromatography by major instrumentation companies.

微型化已成为现代分析仪器发展的一个重要趋势。微型化的分析系统可以有效地减少样品和试剂的消耗,提高检测效率,降低检测成本。作为重要的分离分析手段,色谱仪器的微型化也是分离科学未来发展的重要趋势。从色谱仪器的角度看,微型化可以带来的优势包括^[1,2,3,4,5,6,7]^: (1)溶剂消耗量的大幅减少,理想状态下相较于常规色谱系统可减少溶剂消耗近3个数量级;(2)样品需求量下降,适合生物组学研究等无法获得大量样品的分析领域;(3)快速的分离分析;(4)有利于色谱装置的模块化、集成化设计。此外,在液相色谱-质谱联用中,由于电喷雾离子源(ESI)的离子化效率与前端色谱流速的倒数具有线性关系^[8]^,色谱微型化带来的低流速可以有效适配ESI-MS,适用于分析生物组学研究中常见的微量复杂样品。

基于微流控芯片平台的液相色谱被称为芯片液相色谱。得益于微机电技术(MEMS)强大的微结构加工能力,相较于另一类微型化色谱——毛细管液相色谱,芯片液相色谱具有更高的灵活度和可集成性,在微型化、模块化、智能化、自动化等方面,芯片液相色谱具有更好的发展前景。目前,芯片液相色谱可以良好地实现常规液相色谱的富集、分离等功能^[9,10,11,12,13,14,15,16,17]^,产业界也在芯片液相色谱商品化上取得一定成果。本文将着重介绍近年来芯片液相色谱技术在学术界和产业界的最新进展,并展望芯片液相色谱技术未来的发展方向。

## 1 芯片色谱系统

芯片色谱系统的设计、加工、使用是一个复杂的系统工程。根据具体分离任务的需要,芯片基底材料的选择、色谱固定相的选择、芯片通道结构的设计与制造、流体驱动方式以及芯片连接方式的选择、检测器的选择与联用、特殊色谱结构或方法的联用,各个要素相互影响、相互牵制,每一个要素都具有重要的作用。

### 1.1 芯片基底材料

芯片基底材料的选择需要综合考虑材料的特性(硬度、形变模量、化学惰性、吸光性质、吸脱附性质、生物兼容性等),并根据所拥有的加工手段以及分离分析的具体条件来决定。最早使用的芯片色谱基底材料是硅^[18]^。由于早期微流控芯片加工工艺大部分直接继承自微机电加工技术,硅自然成为工程师们最为熟悉的芯片材料。硅材料具有较高的硬度和良好的化学惰性,适用于绝大多数色谱方法,因此硅在早期芯片液相色谱领域内有很多应用^[19,20,21]^。但是,硅在紫外以及可见光区无法透射,这导致在硅芯片上直接原位使用光学检测较为困难。因此,人们常用玻璃或石英材料替换硅。玻璃以及石英具有出色的化学稳定性、机械强度、优良的生物兼容性和可衍生能力,同时还具有极高的透射率。Belder课题组在玻璃芯片上开展了系统性的工作^[22,23,24]^。他们设计了一整套标准化的玻璃芯片器件(见图1a),这些芯片结合了液相色谱分离与ESI离子源,并配备了高压不锈钢夹具用于芯片与外部设备的连接。这种高压不锈钢夹具可以承受高达36 MPa的流体压力,同时还可以实现极低死体积(约2~10 nL)的侧向芯片连接。Mellors等^[25,26]^设计了一种毛细管电泳芯片并与ESI-MS联用,他们将矩形芯片的一个角直接作为ESI喷口,证明了玻璃芯片可以直接作为ESI离子源的喷口。利用玻璃对高温的耐受性,Heiland等^[27]^开发了具有温度梯度洗脱功能的玻璃色谱芯片,并用于分离多环芳烃。这种温度梯度芯片可在以4 ℃/s的温度梯度升温至200 ℃的梯度条件下工作。该课题组还利用玻璃材料优良的稳定性和机械强度,在同一套玻璃芯片的基础上开发了芯片超临界流体色谱(supercritical fluid chromatography, SFC)联用双光子激发(two-photon excitation, TPE)荧光光谱装置^[28]^。这一装置可在20 s内完成色谱分离,且在20 mm/s的高流速下仍能得到高度对称的色谱峰。

**图 1:**
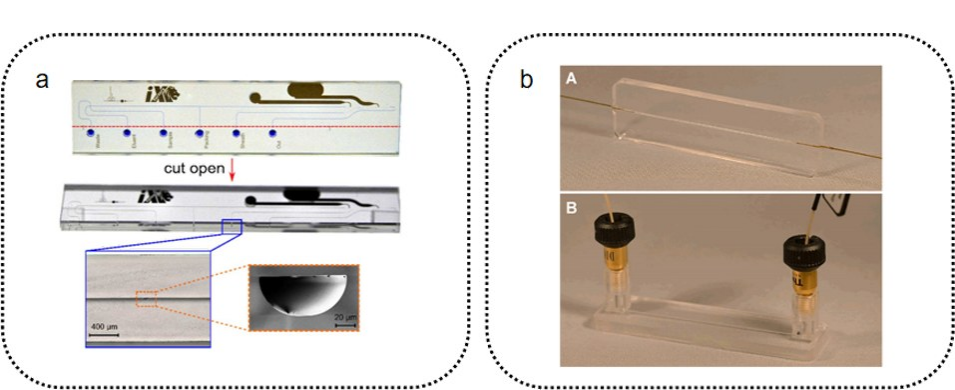
芯片色谱基底材料

与硅和玻璃材料相比,聚合物基底材料具有更良好的加工性能,是目前微流控芯片领域最常用的芯片基底材料。基于聚合物材料,人们已发展了丰富的加工技术,如激光烧灼技术^[29,30]^、软光刻技术^[31]^、喷射造型技术^[4]^等。这些稳定且成熟的物理加工手段使得聚合物芯片具有较高的批次间重现性。然而,聚合物芯片在化学稳定性和机械强度方面,要普遍逊色于石英芯片和玻璃芯片。聚二甲基硅氧烷(PDMS)是目前应用最为广泛的聚合物微流控芯片材料,PDMS具有优良的透光率和生物兼容性。同时,PDMS还极为柔软(弹性模量约为500~4000 kPa),只需要施加一个大气压的压力就可以引起PDMS一个维度上近10%的形变^[32]^。这使得PDMS相比玻璃芯片可以更容易地实现芯片与其他设备的连接,甚至可以在PDMS芯片通道内直接加工泵阀结构^[33]^。但PDMS的缺点亦十分明显^[4]^: PDMS材料在常用的色谱流动相溶剂中易发生溶胀;其较强的吸附性质和透气性会导致较严重的色谱峰展宽;低弹性模量使得PDMS芯片无法承受高流体压力,不适合高效液相色谱这类流体背压较大的色谱方法。以上诸多问题使得PDMS材料在芯片色谱领域内的应用受到较大限制。但PDMS作为极易加工和批量生产的芯片材料,在微流控领域常作为原型设计使用。热塑性材料(thermoplastics)的高分子链结构更加紧密,在加热到玻璃化转变温度时热塑性材料会由固态转变为具有一定流动性的状态,其冷却后会固定形态的性质称为热塑性。热塑性的芯片材料有^[34]^:聚碳酸酯(PC)、聚甲基丙烯酸酯(PMMA)、环烯烃共聚物(copolymers of cycloolefin, COC)等。相比于以PDMS为代表的弹性体(elastomer),热塑性材料具有更高的机械强度(如PMMA弹性模量可达3.2 GPa^[35]^)、更强的抗溶剂腐蚀性能和更优良的可加工性。Wouters等^[36]^开发了基于COC的高效液相色谱芯片系统(见图1b),他们利用微铣削技术直接在COC基底上铣出芯片通道,通过溶剂-真空辅助键合技术封装芯片,配合特别设计的芯片接口制成在38 MPa压力下可长时间运行的COC液相色谱芯片,并利用这一系统完成了烷基苯酮的快速分离。还有一些特殊的芯片基底材料,如钛^[37,38]^、陶瓷^[39]^、钻石^[40]^等,基于它们极高的机械强度、极强的耐腐蚀性能、极高的热稳定性等原因,也被尝试应用于芯片色谱基底材料。但由于它们较为苛刻的加工和制造条件,目前这几类芯片基底材料还没有被大规模的应用。

### 1.2 芯片色谱柱

最早投入使用的芯片色谱柱结构是开管柱(open-tube)^[18,41]^。开管柱床的制备是在色谱柱通道内壁上修饰硅烷、凝胶、聚合物等作为固定相。开管柱因其中空的柱床结构而具有最小的分离阻抗,开管柱床可以相对容易地在芯片孔道内实现。但其中空的结构也导致开管柱的柱床比表面积低,相比低,色谱柱容量小。提高开管柱柱容量的关键是提高固定相层的比表面积。Collins等^[42]^利用光引发聚合反应在毛细管上可控地生成聚合物开管柱床(见图2a),所获得柱床厚度的相对标准偏差在±0.8%,实现了高度可控的开管柱床制备。Yang等^[43]^在制备柱床时,将聚合物前体与商品化的色谱填料颗粒混合注入刻蚀好的柱管,之后用紫外光引发聚合反应。生成的聚合物层将填料颗粒包裹并固定在管壁上形成柱床。这些工作在一定程度上改善了开管柱的上样量,但仍无法从根本上解决开管柱的柱容量问题。

**图 2:**
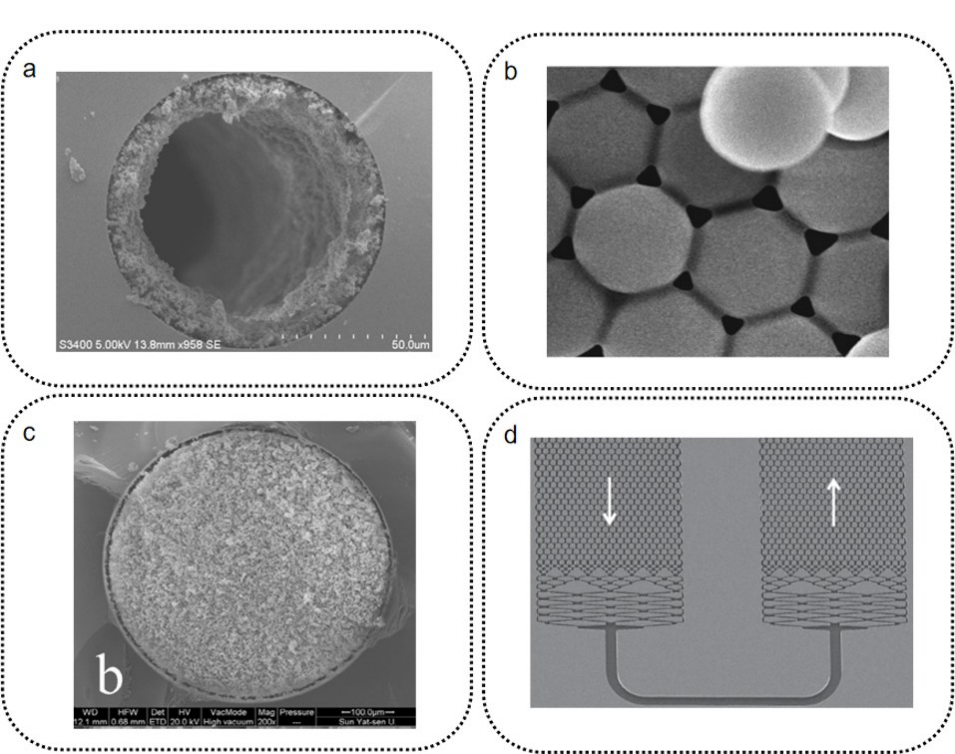
芯片色谱柱床电子显微镜照片

填充柱床(packed bed)是将预先制备好的固定相填料颗粒通过流体带动填装进色谱柱管形成的色谱柱床。填充柱具有显著优于开管柱的比表面积。由于柱管内充满填料颗粒,填充柱流体背压通常可以达到5~40 MPa。得益于色谱固定相技术数十年间的发展,填充柱拥有种类和功能都十分丰富的色谱固定相库可供选择。在微流控芯片上制备填充床需要解决两个问题:(1)如何将填充颗粒固定在芯片孔道内;(2)如何提高填料装填的重现性。固定填料颗粒常用的方法是在微流控芯片通道内构建柱塞(frit)结构。柱塞结构可以通过微加工手段制得,也可以利用原位聚合反应制备聚合物柱塞。Thurmann等^[44]^利用激光辅助的光聚合手段在芯片通道中原位聚合生成一段约100 μm长的多孔聚合物整体柱塞。之后进行颗粒填料装填形成色谱柱床,最后在柱床末端再聚合制备一段柱塞完成色谱柱床两头的固定。这种不到100 μm长的多孔聚合物整体柱塞能承受25 MPa的装填压力,可以很好地满足芯片柱装填和使用的承压要求。Huft等^[45,46]^利用PDMS的柔性,设计了一种多层PDMS多柱液相色谱芯片,并应用于免疫球蛋白基因逆转录PCR扩增产物的分离纯化。这一芯片通过微阀挤压芯片孔道形成局部锥形结构,利用基石效应(keystone effect)^[47]^将色谱填料固定在柱管内。同时,他们还在芯片柱管壁上加工了大量微型旁路阵列,极大地减小了装填时的阻力,可有效辅助填料在PDMS孔道内的装填。旁路阵列在进行色谱分离时会通过微波照射封闭,不影响正常的色谱分离。本课题组的单颗粒柱塞技术^[48]^也被运用到芯片填充柱的制备上。单颗粒柱塞是一种多孔的硅球颗粒,其颗粒尺寸略大于分离通道尺寸,此前常用于各类毛细管色谱柱的制备。使用时,只需将单颗粒直接塞入通道即可形成柱塞,使用方便可靠。Li等^[49]^将多孔的单颗粒柱塞安装进入芯片通道内用于固定填充柱床,制备好的芯片柱可在约34.4 MPa的高压下工作。该芯片可在5 min内完成3种单胺类神经递质的分离,理论塔板数达到100000块/米。柱塞结构并非固定柱床的唯一方法,另一种方法是基于纳米微球颗粒自组装形成填充柱床。Shaabani等^[50]^将甲基丙烯酸-2-羟基乙酯(HEMA)和二甲基丙烯酸乙二醇酯(EDMA)、光引发剂、致孔剂以及二氧化硅纳米颗粒混合后注入PDMS芯片的通道。通过光引发使HEMA和EDMA聚合,聚合过程中二氧化硅纳米颗粒发生自组装相互交联形成胶体自组装(colloidal self-assemble, CSA)柱床(见图2b)。该芯片在7天时间内进行28次蛋白质分离实验,蛋白质的洗脱时间相对标准偏差小于0.83%,芯片分离能力稳定。同一蛋白质片段在不同芯片(3片芯片)间洗脱时间的相对标准偏差为4.3%,芯片间也有较好的重现性。纳米填料颗粒自组装形成的柱床具有较高的重现性,且对装柱技术要求较低。但由于纳米颗粒粒径极小,导致柱背压极大,因此除了电色谱方法外,其他液相色谱方法难以在纳米颗粒自组装柱床上使用。

整体柱床(monolithic bed)是一种通过聚合反应在色谱柱管内原位合成的固定相结构。相比于填充柱^[51]^,整体柱的流阻较小,且不需要柱塞结构来固定柱床。相比于开管柱,整体柱具有更高的比表面积和柱容量。其原位聚合的模式也使整体柱在芯片色谱领域的应用备受关注。整体柱床主要的缺陷是^[2]^: (1)聚合反应可控性较差,柱间重现性较难保证;(2)聚合反应的化学环境对芯片材料的选择有限制;(3)在聚合反应前后,整体柱床的尺寸常发生一定“缩水”,容易导致柱床脱落。Kendall等^[52]^开发了一种非原位的整体柱床制备方法。他们首先在一个芯片模具中制备整体柱床,之后拆开模具芯片并取出柱床。在对柱床做衍生化处理后,再将其放入一个通道尺寸缩小10%的芯片,最后封闭芯片完成制备。这种制备方式虽然失去了整体柱原位聚合的优点,但换来的是尺寸更加可控的整体柱床制备。在解决柱床收缩问题的同时,还可以实现不同功能化的柱床联用,实现多维分离。除了常见的有机聚合物整体柱床,无机材料也被应用于芯片整体柱的制备:Zhai等^[53]^开发了一种基于氧化石墨烯硅烷聚合物的分子印迹整体材料芯片色谱柱(见图2c),并用于分离富集辣椒粉中的罗丹明B。他们将氧化石墨烯(GO)与3-氨丙基三乙氧基硅烷耦联生成氧化石墨烯硅烷(GO/SiO_2_),之后通过交联剂乙二醇二甲基丙烯酸酯(EDMA)与GO/SiO_2_反应生成整体柱床。该芯片色谱柱对罗丹明B富集因子可达110,检出限为0.40 ng/g。

柱阵列柱床(pillar array bed)是芯片色谱独有的柱床结构。柱阵列柱床的原型是一种被称为COMOSS(collocated monolithic support structure)的微流控芯片结构^[54,55]^。这种结构由大量规则排列的微柱组成,通常是通过光刻结合深反应离子刻蚀(DRIE)技术加工制成。基于物理加工手段的制造方法使得柱阵列柱床具有极高的重现性,并且可以批量复制。单纯的COMOSS结构比表面积较低,样品载量低,作为色谱柱床使用需要对微柱阵列进行额外的修饰。Lincoln等^[56]^研究了在柱阵列表面修饰多孔层的厚度对柱效以及保留时间的影响。多孔层厚度的增加对理论塔板数的提升较小,但保留性能会有明显的增加。同时,极大提高的比表面积也对柱阵列柱床的载样能力有显著提升。Desmet课题组对柱阵列色谱(pillar array chromatography, PAC)技术的成熟完善以及应用做了较为系统的工作^[57,58,59,60]^(见图2d),包括:柱阵列色谱柱的制备、流体动力学模型、柱阵列多孔层研究、柱阵列长柱制备等方面。尤其在柱阵列芯片色谱长柱制备上,Desmet课题组做出了系统性的工作。Baca等^[61]^利用串联4张柱长为2 m的柱阵列色谱芯片得到的8 m长的柱阵列色谱柱分离小分子混合物,在2050 min的梯度时间内得到了1815的峰容量。由于柱阵列的结构特点,相比于8 m的柱长,该串联芯片组可以在较低的流体压力(25 MPa)下以0.60 mm/s的线速度完成分离。

### 1.3 流体控制

芯片色谱是以微流控芯片作为载体的色谱系统,其核心-微流控芯片的关键功能就在于流体控制。高效的微流体控制是芯片色谱正常工作的基础。进样是色谱分析极为重要的一个步骤。进样量过大会导致进样时间过长、色谱柱过载、峰展宽等问题,严重影响色谱分离的效率。目前与高效液相色谱配套的进样针的规格普遍在微升级别,而芯片色谱平台需要控制纳升级别的进样量。因此,开发与芯片色谱相匹配的进样系统显得十分重要。芯片色谱进样技术大致可分为电动进样与压力进样两类。其中,电动进样无需泵阀结构、操作简单的特点使其成为最早投入使用的方法(T型和双T型电动进样通道)^[62,63]^,同时也是目前最常用的芯片色谱进样模式。Cong等^[64]^开发了一种电动阀门式的芯片色谱进样结构(见图3a)。这种结构中有一个电驱动的可变型阀门,阀门两侧分别是进样通道和色谱柱。在准备阶段,在样品通道与色谱通道两端施加电压,样品先进入样品通道,并由于阀门阻挡无法进入色谱柱。在进样阶段,阀门脉冲开关打开,样品定量进入色谱通道,之后阀门关闭并开始色谱分离。电动进样由于扩散、迁移率等问题^[65]^,通常进样误差很大,很难做到定量进样。相对的,基于机械手段推动样品的压力进样方式可以实现高精度的定量进样。但由于需要对流体额外施加压力,压力进样需要更多泵阀结构提供支持(如Agilent HPLC-chip的3层六通阀结构^[66]^)。压力进样也可以通过分流的操作方式摆脱泵阀结构,从而实现简单的定量进样。Gáspár等^[67]^在PDMS芯片上设计了一种分流进样结构,由进样口注入分流区的样品(微升级)会依据分流结构各通道的宽度比进行分流。通过控制进入色谱柱的通道与其他分流通道的比例就可以将进样量精确地控制在纳升级。但需要指出,分流式压力进样需要较大的样品量,且不可避免地会造成样品浪费。Ha等^[68]^开发了一种特殊的“穿刺”进样方法,他们直接将微升级的进样针扎入PDMS芯片的通道中,之后用千分尺调节进样针筒活塞位置实现“穿刺进样”。由于千分尺精确的距离调节,这种进样方式可实现3 nL体积的精准可重复进样,且非常廉价、使用简单。

**图 3:**
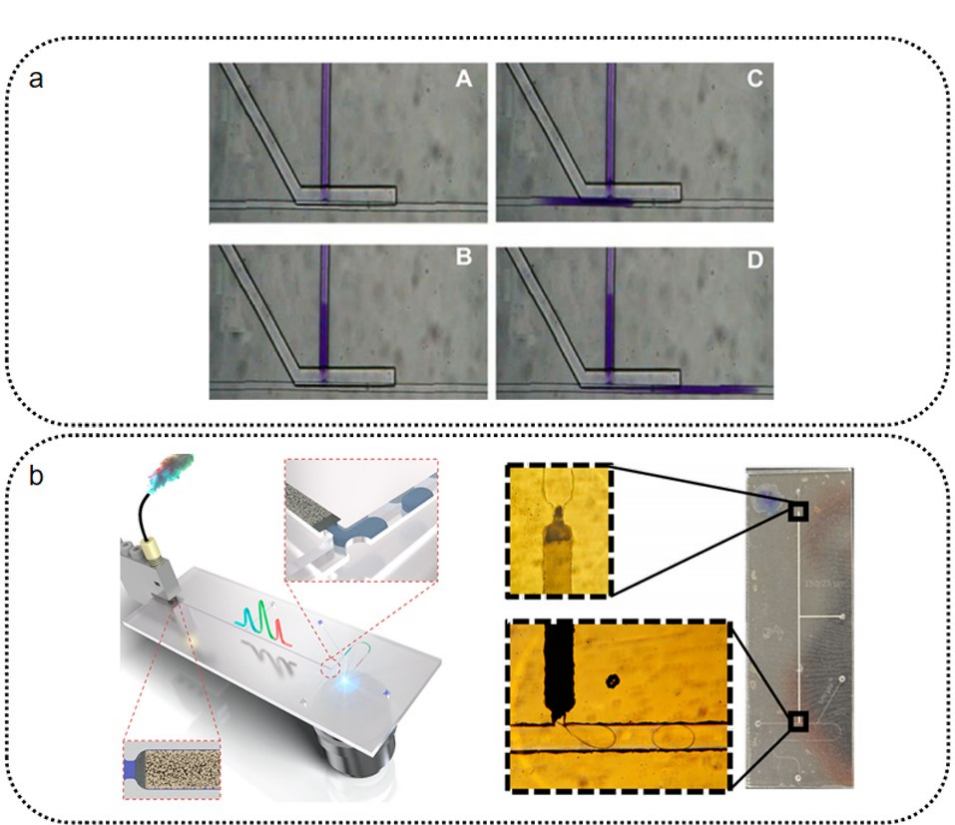
芯片色谱流体控制措施

芯片色谱泵可简单分为芯片外置泵和芯片内置泵。早期芯片色谱泵直接沿用高效液相色谱设备使用的常规高压泵。但由于芯片通道尺寸减小,这些常规泵在进行梯度分离时会发生严重的梯度滞后现象。因此,常规泵需要加入分流装置来调整流速,以适应芯片色谱的工作条件^[69,70]^。但分流在梯度分离中会造成严重的溶剂浪费^[71]^,因此芯片色谱需要与其匹配的低流速高压泵系统。在芯片外置泵中,能够同时实现低流速和高压力的最简单的泵类型就是注射泵。Grinias等^[72]^通过阀门切换梯度储存流路与UPLC泵和气动放大泵的连接实现了一种高压注射泵结构。在准备阶段,常规UPLC泵与梯度储存流路相连,配制好的流动相被注入梯度储存流路并保存在其中。在色谱分离阶段,切换阀门使装载有流动相的梯度储存流路与色谱柱相连,启动与梯度储存流路相连的气动泵,使储存的流动相以一个较高的压力注入色谱柱中完成分离。这一泵结构可以达到300 MPa的运行压力。芯片内置泵的驱动方式以电驱动、电渗流和磁驱动为主,虽然也存在芯片内置机械泵,但蠕动泵、气动泵结构在芯片上的加工难度较高,应用实例很少。最具代表性的芯片内置泵是电渗泵(electroosmotic pumps, EOP)。EOP有如下几点优势^[73]^: (1)可直接集成到微流控芯片上;(2)可生成无脉冲液流;(3)可快速改变液流大小和方向;(4)没有运动组件,可有效提高泵的稳定性和寿命;(5)在较大的背压范围内都可产生中低流速液流。Wang等^[74]^开发了一种高压EOP芯片,该芯片泵的工作压力约17 MPa,工作流速约为500 nL/min,该条件已足以支持芯片液相色谱的运行。该芯片由大量EOP基本单元组成,每个EOP基本单元由数个电渗通道并联而成。每个基本单元再与其他单元串联成整个EOP芯片,芯片泵可产生的液流压力直接正比于芯片内含的EOP基本单元数量。

液滴技术作为一种新型的微流体控制技术也被应用到了芯片色谱上。Gerhardt等^[75]^将液滴微流控技术与HPLC芯片无缝组合(见图3b),在HPLC芯片色谱柱出口处耦合T型液滴生成通道。利用与色谱通道正交的油相切割水相色谱洗脱物,将洗脱物以45 Hz的频率切割为大量体积约1 nL的液滴。静态液滴内流体为层流状态,具有低扩散、无返混的特点。因此将色谱洗脱物切割为液滴的过程,相当于将色谱分离的色谱图谱切割成大量片段并进行保存,防止洗脱物返混合污染,最大限度地保留了分离分辨率。这一利用液滴保存色谱结果的技术也为洗脱物在柱后进一步的处理与分析提供了时间和空间条件。

### 1.4 检测器

相较于常规色谱,芯片色谱需要更加灵敏、响应更快的检测器。芯片色谱常用的检测器可分为光学检测器、电化学检测器、质谱检测器3类。紫外-可见光谱法是分析化学中应用最为广泛的光学检测手段,其简单的结构使其在芯片色谱中有着广泛的应用。但芯片通道尺寸相较常规色谱柱径的大幅度缩小,使得芯片色谱通道的光程大幅缩小。这极大影响了紫外-可见光谱在芯片色谱上原位检测的灵敏度^[2,4]^(尤其是当通道宽度小于100 μm时)。因此,在芯片色谱上进行紫外-可见光谱检测需要通过增大通道宽度或添加微结构(如光纤^[76]^、波导管^[77]^)等手段来增加光程,以提高检测灵敏度。相比之下,荧光光谱的发射光强度与激发光强度成正相关,在使用激光这样的强光源作为激发光源时(激光诱导荧光光谱,LIF),光程的减小对荧光光谱检测灵敏度的影响几乎可以忽略^[78]^。荧光光谱主要局限于可自发荧光的物种有限,目前需要借助荧光标记和荧光染料才能做到广泛应用。TPE荧光光谱利用两个光子同时激发一个分子,可使分子达到更高的能级,从而让一些原本不会发出荧光的分子产生荧光信号,该技术有望实现无标记的广泛荧光检测。Hackl等^[79]^将电色谱芯片与TPE荧光光谱联用(见图4a),实现了芯片色谱平台上的无标记时间分辨荧光光谱检测。该芯片对多环芳烃的检测灵敏度达到了nmol级。他们同时还证明了532 nm的TPE荧光光谱可以达到与266 nm单光子激发(one-photon excitation, OPE)荧光光谱相近的灵敏度。这意味着双光子激发荧光光谱可在聚合物芯片等具有一定紫外吸收能力、但更容易制作的芯片平台上使用,而无标记单光子激发荧光光谱则需要使用石英等低紫外吸收的芯片材料来保证检测灵敏度。除了紫外-可见光谱、荧光光谱外,拉曼光谱(相干反斯托克拉曼散射(coherent anti-Stokes Raman scattering, CASR^[80]^)、表面增强拉曼光谱(surface-enhanced raman spectroscopy, SERS^[81]^))也被尝试应用于芯片色谱检测器,并表现出可期的前景。

**图 4:**
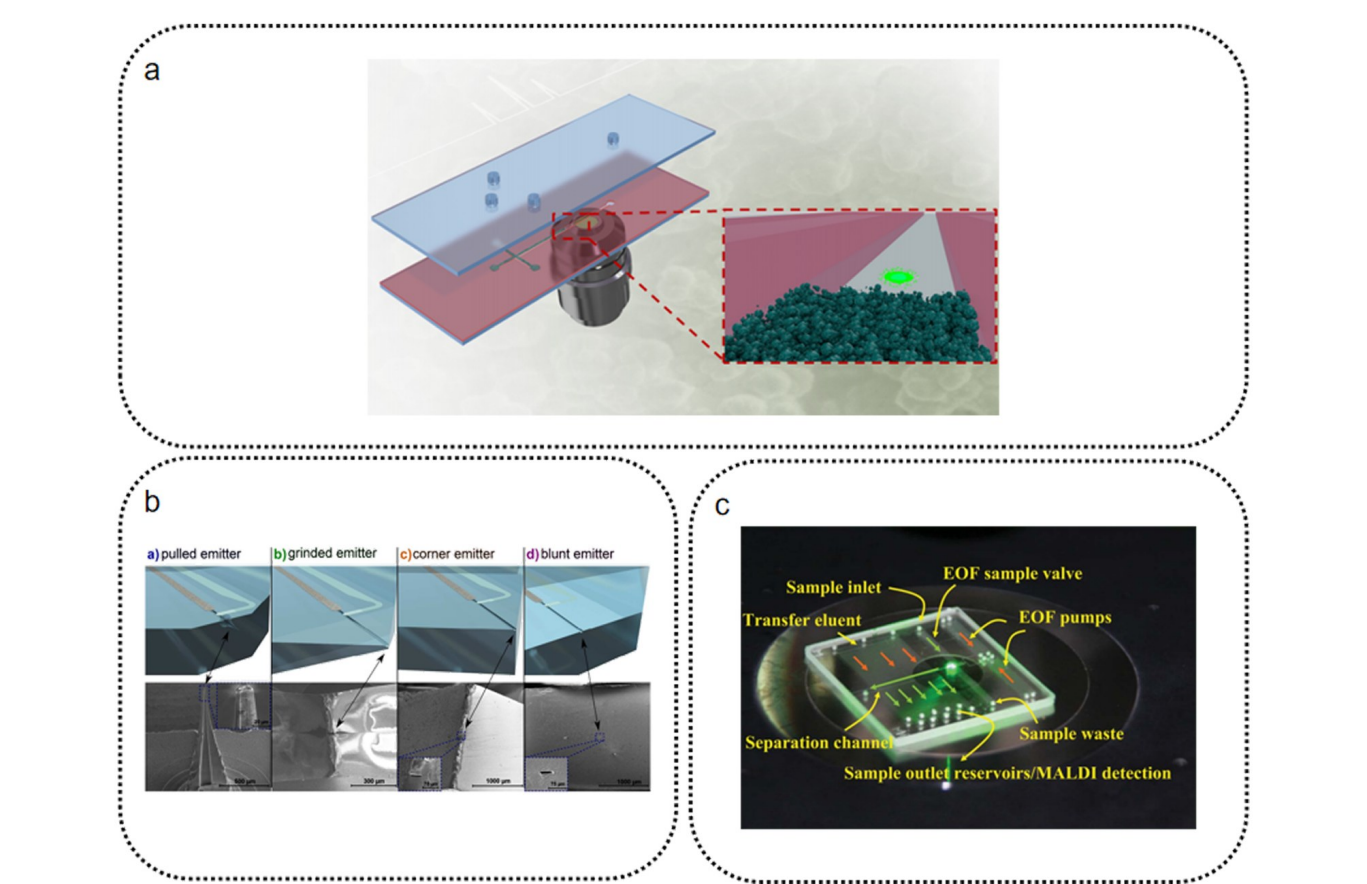
部分芯片色谱检测器

电化学检测器是通过检测电极表面电化学反应产生的电信号(电流、电压)来进行分析检测的手段。用于电化学检测的电极可以很容易地进行微型化,并集成到微流控芯片平台上。而且,电化学检测的工作站已经可以做到小型化便携式,因此电化学检测方法也被视为实现便携式芯片色谱的重要手段^[82,83]^。电化学检测方法在芯片色谱中的应用有一个限制因素:电极的使用寿命有限,需要经常更换;常见的微流控芯片在封装后就无法再拆开,因此电极损坏后检测芯片就只能报废处理。Erkal等^[84]^为解决这一问题,开发了一种3D打印的电化学检测芯片。这种芯片在电极位置加工了螺纹结构用于可替换电极的固定;同时螺纹结构还满足了芯片的密封需求,使得芯片多次更换电极也不会漏液。相比于常规电导、电势检测需要的电极与溶液直接接触,电容耦合非接触电导检测(capacitively coupled contactless conductivity detection, C^4^D)不需要与待测溶液直接接触,且电导检测灵敏度极高,其作为微流控芯片检测器更具潜力^[85,86]^。Beutner等^[87]^设计了一种C^4^D与质谱联用的检测方法,用于毛细管电泳分析检测酚类物质。他们发现这两种检测方法具有较好的互补性:C^4^D检测器对间甲酚具有极佳的灵敏度而对硝基酚敏感性不佳,而质谱检测器则正好相反。

质谱法是基于不同质荷比的离子在电场加速后进入磁场中运动轨迹的不同,或在真空中飞行时间的不同,进行物质鉴定的检测方法。质谱法的质量分辨本领使其具有极高的灵敏度和分辨率,以及超快的分析速度,同时还能提供丰富的分析信息。相比于光学检测器和电化学检测器,质谱检测器显然是三者之中结构最为复杂,成本最为昂贵的检测器。但由于质谱法极高的灵敏度、微型化色谱与质谱极好的相容性以及质谱在组学研究中的重要地位,芯片色谱与质谱的联用仍然是前沿的研究方向^[88]^。芯片色谱与质谱联用需要解决色谱出口与质谱离子源之间的耦合问题。目前常用的芯片色谱-质谱联用离子源是ESI和基质辅助激光解吸电离离子源(matrix-assisted laser desorption/ionization, MALDI)。ESI离子源因其广泛的应用性和高灵敏度而成为与芯片色谱耦合最理想的离子源。Lotter等^[23]^对玻璃芯片的ESI喷口进行了较为系统的探究。他们研究了4类ESI喷口形状对质谱检测效果的影响(见图4b),发现在高流速(约400 nL/min)条件下4种芯片喷口没有明显的区别。但是在低流速(<50 nL/min)条件下,尖锐的ESI喷口(pulled和ground型)产生的电喷雾更加稳定,离子化效果更好。加工芯片ESI喷口需要较为精密的加工技术,一种更为简单的喷口构建方法是在芯片柱上嵌入一段拉尖毛细管作为ESI喷口。Dietze等^[89]^在制作聚合物芯片时,在通道中嵌入一段一端烧蚀拉尖的毛细管,成功与ESI源耦合。这种利用毛细管针尖制作喷口的方法特别适合于聚合物这类无法直接作为ESI喷嘴的芯片基底材料。MALDI离子源应用于微流控芯片上,可分为在线离子源^[90]^和离线离子源^[91]^两类。人们已报道了数类微流控MALDI芯片^[92]^,但带有色谱分离功能的芯片色谱-MALDI-MS较少。Lazar等^[93]^开发了一种新型的液相色谱-MALDI-MS芯片(见图4c),该芯片以C18颗粒填料作为色谱固定相,在色谱柱通道的正交方向制作了大量与色谱柱通道相通的MALDI收集通道。在进样并完成色谱分离后,洗脱物不再由色谱柱轴向洗脱,而是通过与MALDI收集通道相连的电渗泵结构横向泵入MALDI储液槽中,直接进行MALDI-MS检测。由于MALDI收集通道被集成在色谱柱通道侧向,进行洗脱物收集时相当于对色谱图进行了切割和分段检测,这使得该系统可以获得优良的分辨率和检测通量。

## 2 芯片色谱的商品化

2005年,Yin等^[94]^报道了安捷伦(Agilent)公司HPLC-Chip/MS系统的原型,标志着芯片色谱的发展正式进入商品化阶段。商品化的芯片色谱系统需要做到坚实耐用、高强健性以及不逊色于常规色谱系统的分离能力。为了做到使用方便简单,商品化芯片色谱系统通常还需要完备的配套系统,包括精密的芯片-外设连接、稳定的流体驱动、灵敏的检测器等。本部分将简要介绍4家公司的芯片色谱产品以及这些产品的实际应用情况。

### 2.1 安捷伦

Agilent HPLC-Chip/MS系统是安捷伦公司的经典芯片色谱产品,也是最早投入实际应用的芯片色谱系统。该色谱芯片由激光烧灼的聚酰亚胺作为基底材料制成,包含多层结构。芯片内含:富集柱(40/80 nL,填充5 μm Zorbax 300SB C18填料)、分析柱(75 μm×150 mm,填充5 μm Zorbax 300SB C18填料)、ESI喷口、多层六通阀进样系统,芯片结构和填料可在一定程度上定制。芯片配套Chip-Cube系统实现芯片与各外部设备的标准管路连接。Chip-Cube系统的转子可切换芯片六通阀的连接状态实现进样、样品富集、样品分离等操作。该芯片系统的应用主要集中在蛋白质定量^[94,95]^、糖蛋白分析^[96]^、蛋白质磷酸化分析^[97]^、药用小分子分离^[98]^等领域。Bishop等^[99]^报道了安捷伦HPLC-Chip/MS系统适配电感耦合等离子体(ICP)离子源的改良版本,新系统去除了原本的ESI喷口,而通过Chip-Cube将分析柱通向特制的气动喷雾器来联用ICP-MS。该系统被用于分离检测马血浆中的维生素B12, 7次平行试验中,维生素B12保留时间的相对标准偏差为0.19%,检出限为14 ng/mL。

### 2.2 Sciex-Eksigent

cHiPLC系统是Eksigent公司开发的芯片色谱系统。Sciex公司收购Eksigent公司液相色谱产品线后,cHiPLC系统仍被冠名为Eksigent-cHiPLC。cHiPLC系统可同时操作3张石英液相色谱芯片,每张芯片都可以具有不同的功能(富集、分离等)且可以相互串联,这使得cHiPLC芯片间的组合可实现多种色谱工作模式。cHiPLC分析柱有两种规格:nano-cHiPLC(75 μm×150 mm)和micro-cHiPLC(200 μm×150 mm)。芯片柱使用圆形截面通道,围堰固定填充柱床,以及数种可选择的固定相填料。其芯片结构可支持高压流体运行,且连接死体积极小(约13 nL)。cHiPLC系统的双分析柱模式可以实现两张芯片色谱的交替分离,一张芯片色谱在进行分离的同时可以对另一张芯片进行冲洗,这种工作模式可以减少色谱柱的准备时间,提高分析效率。cHiPLC系统主要的应用领域有:蛋白质组学^[100, 101]^、糖肽分析^[102]^、酶活性测试^[103]^等。

### 2.3 沃特世(Waters)

沃特世目前有两款芯片色谱产品:ionKey/MS系统和TRIZAIC nanotile,两种色谱芯片所使用的的基底材料均为陶瓷材料。ionKey/MS芯片为150 μm内径填充柱(填充沃特世亚2 μm填料,UPLC分离)液相色谱芯片。该芯片通过沃特世的iKey结构实现与外部设备的连接。该结构完全取代传统的色谱接口,可以实现“即插即用(plug and play)”,最大限度地消除了操作人员的技术水平差异。该芯片柱还可以配备柱后添加通道(pass column adding, PCA),对色谱分离物添加特定试剂,辅助后续分析操作。ionKey/MS系统主要应用于蛋白质组学研究^[104,105]^。TRIZAIC nanotile芯片可作为富集芯片(180 μm×20 mm,填充5 μm C18填料)或分析芯片(180 μm×100 mm,填充1.8 μm HSS T3填料颗粒)使用,主要的应用有类固醇分析^[106]^和人血清多肽定量分析^[107]^。

### 2.4 PharmaFluidics

PharmaFluidics是芯片色谱领域一家年轻的公司,成立于2010年,其在2017年推出μPAC柱阵列色谱芯片系列。目前有两种芯片规格:200 cm μPAC芯片和50 cm μPAC芯片。μPAC芯片采用Si-Pyrex glass基底材料,固定相采用5 μm直径的圆柱柱阵列柱床(柱间距2.5 μm)。柱阵列柱床结构使200 cm μPAC芯片可以在1.5 μL/min的高流动相流速下以35 MPa的压力运行。柱阵列柱床也使该芯片产品具有良好的重现性和稳定性。μPAC芯片没有配套外设系统,芯片通道通过环氧胶粘剂连接标准石英毛细管。芯片可通过毛细管与任何带有标准色谱PEEK接头的设备进行连接,这在一定程度上增加了μPAC芯片的使用难度,但也提高了该芯片设备搭建的自由度,使其更适合科研实验室使用。作为较新的芯片色谱产品,μPAC芯片目前应用实例较少。Mann课题组^[108]^将其用于蛋白质组学研究,基于μPAC芯片搭建的LC-MS/MS平台鉴定了340000个蛋白质,将此前蛋白组学严格分辨的蛋白质数量翻倍,为蛋白质组学数据库的丰富做出了卓越贡献。

## 3 总结与展望

从历史上第一个芯片色谱装置诞生至今已近30年,但芯片色谱技术仍基本停留在基础研究和科研实验室里。相较于最初的期待,芯片色谱走向产业化的推进速度显得些许缓慢。目前存在的主要瓶颈在于:芯片色谱技术对微型化、集成化的需求与芯片材料、工艺和设计发展现状的矛盾。微流控芯片微型化、集成化主要通过缩小通道尺寸和增加通道总长度实现。对微纳尺度流体,通道尺寸的缩小和长度的增加都会大幅增加流体阻力。现有的芯片基底材料承压能力普遍在60 MPa以下,材料性质限制了色谱芯片微型化和集成化的进一步提升。这一瓶颈的解决在于发展新型的芯片基底材料,尤其是高强度和优良加工性的聚合物材料;同时也需要产业界形成相对统一标准的流体通道基本结构设计,以提高材料性能的利用度。此外,与芯片色谱匹配的外部设备微型化程度低,芯片接口技术尚不成熟,以及芯片色谱产业缺乏统一行业标准等,都有待学术界与产业界共同努力解决。但客观地看,色谱微型化的趋势已经十分明显:毛细管色谱的产业化、微流甚至纳流色谱的推广及其在生物医学分析中应用已日渐增多。作为色谱微型化另一途径的芯片色谱,正式走向实际应用只是时间问题。芯片液相色谱极高的可扩展性、可集成性以及模块化的优势使其最有可能成为色谱这项技术走入便携检测(POCT)领域的方式,但这一理想还有待进一步提高芯片集成度和系统微型化程度才能真正实现。相信在不久的将来,“plug-and-play”的芯片色谱将成为现实。
